# Beneficial effects of a novel polyherbal formulation on the skeletal muscle antioxidant status, inflammation, and muscle-signaling proteins in exercised rats

**DOI:** 10.55730/1300-0152.2682

**Published:** 2023-12-14

**Authors:** Mehmet TUZCU, Oğuzhan ÖZDEMİR, Cemal ORHAN, Nurhan ŞAHİN, Abhijeet MORDE, Muralidhara PADIGARU, Prakash BHANUSE, Kazım ŞAHİN

**Affiliations:** 1 Department of Biology, Faculty of Science, Firat University, Elazığ, Turkiye; 2 Department of Veterinary Science, Technical Sciences Vocational School, Batman University, Batman, Turkiye; 3 Department of Animal Nutrition, Faculty of Veterinary Medicine, Firat University, Elazığ, Turkiye; 4 OmniActive Health Technologies, Lower Parel, Mumbai, India

**Keywords:** Joint health formula, inflammation, exhaustion, exercise, antioxidant

## Abstract

**Background/aim:**

Exhausting exercise can damage muscle tissue due to free radical interactions. It is hypothesized that the increase in free radicals following muscle injury, either due to oxidative damage to biomolecules or the activation of inflammatory cytokines, may lead to secondary muscle damage. This study investigated the effects of a novel joint health formula (JHF) containing bisdemethoxycurcumin-enriched curcumin, 3-O-Acetyl-11-keto-beta-boswellic acid-enriched Boswellia (AKBA), and Ashwagandha on exhaustion time, grip strength, antioxidant status, and muscle-signaling proteins in exhaustively exercised rats.

**Materials and methods:**

Twenty-eight rats were divided into four groups: Control (C), exercise (E), E + JHF 100 (100 mg/kg), and E + JHF 200 (200 mg/kg).

**Results:**

An increase in time to exhaustion and grip strength was recorded with JHF supplementation in a dose-dependent manner (p < 0.0001). In addition, serum and muscle lactate dehydrogenase, malondialdehyde, myoglobin, creatine kinase, and lactic acid concentrations were decreased in the groups supplemented with JHF, particularly at the high dose of JHF (200 mg/kg) (p < 0.0001 for all). JHF supplementation also increased antioxidant enzyme activities and suppressed the production of inflammatory cytokines compared to the exercise group (p < 0.0001). Moreover, JHF reduced the levels of PGC-1α, p-70S6K1, MAFbx, MuRF1, and p-mTOR proteins in muscle tissue compared to the exercise group (p < 0.05), being more effective at high doses.

**Conclusion:**

These findings show that JHF might reduce muscle damage by modulating antiinflammatory, antioxidant, and muscle mass regulatory pathways in exhausted training rats. At the same time, JHF improved exercise performance and grip strength.

## 1. Introduction

The beneficial effects of regular, no-exhaustive physical exercise have been known and studied for a long time (Ketelhut and Ketelhut, 2020). Regular physical activity can help avoid chronic conditions such as diabetes, cancer, cardiovascular disease, hypertension, obesity, osteoporosis, and depression. However, the beneficial effects of exercise are lost with fatigue (Adams and Linke, 2019). According to research, high-intensity and/or extended exercise can harm muscle tissue. It has been suggested that the rise of free radical levels after muscle damage directly or indirectly causes secondary muscle damage as biomolecules are harmed by oxidation and inflammatory cytokines (Nobari et al., 2021).

Reactive oxygen species (ROS) generation and antioxidant enzyme modifications are influenced by physical fitness levels and can result in varying degrees of oxidative damage and lipid peroxidation (Zhang et al., 2021a). Oxidative stress is associated with fatigue and impaired muscle and cellular function (Mariacher et al., 2014). As the first line of defense, the antioxidant system can inhibit the production of free radicals and ROS mainly through antioxidant enzymes, including catalase (CAT), superoxide dismutase (SOD), and glutathione peroxidase (GSH-Px) (Liu et al., 2021).

Many studies have assessed the validity of dietary strategies and micronutrients to reduce exercise-induced oxidative stress (Sureda et al., 2014). Some medicinal plants containing antioxidants and antifatigue properties can promote exercise ability and prevent disease (Zhang et al., 2021b). *Curcuma longa*, known as the Indian spice obtained from plant rhizomes, has a long history of use in traditional medicine for treating inflammatory diseases (Jurenka, 2009). Curcuminoids generally contain three main compounds: curcumin, demethoxycurcumin, and bisdemethoxycurcumin (BDMC). Curcumin has been shown to reduce oxidative stress and have mitochondrial protective properties in various animal models, including a lipopolysaccharide-induced animal model (Zhang et al., 2021b). In addition, curcumin supplementation may have an antioxidative effect after exercise by modulating proinflammatory cytokines like tumor necrosis factor-alpha (TNF-α), interleukin-6 (IL-6), and interleukin-8 (IL-8) (Fernández Lázaro et al., 2020). There are two main active compounds of gum resin extract of the *Boswellia serrata* (BSE) plant: pharmacological 11-keto-β-boswellic acid and 3-O-acetyl-11-keto-β-boswellic acid (AKBA). BSE has been demonsrated to have antiinflammatory properties that inhibit 5-lipoxygenase (5-LOX), matrix metalloproteinase-3 (MMP-3), and TNF-α (Henrotin and Mobasheri, 2018). *Withania somnifera*, known as Ashwagandha, is widely used in Indian traditional medicine. It has been reported to have various pharmacological properties, with antitumor, antiinflammatory, antioxidant, immunomodulatory, and antineuropsychiatric disease effects (Speers et al., 2021).

In a recent study on exercising athletes, curcumin and BSE supplements improved plasma levels of oxidative stress, inflammation, and glycation markers (Chilelli et al., 2016). However, research has not determined how these plant component mixtures affect muscle metabolism. Joint health formula (JHF), a new formulation derived from a blend of herbal ingredients, could be a safe and effective nutritional supplement for muscle activation and health. This study was conducted to investigate the possible effects of JHF on muscle endurance capacity and activation in addition to examining the impact of JHF on muscle metabolism, antioxidant ability, antiinflammatory influence, endurance, and power capacities in rats following exhausting exercise.

## 2. Materials and methods

### 2.1. Animal materials

Twenty-eight male 8-week-old Wistar albino rats (220 ± 20 g) were housed in polypropylene cages (12 dark:12 light, 22 °C). Access to water and normal rat food was provided ad libitum. Animal procedures were approved by the Animal Ethics Committee of Bingöl University (2020-04-03) in full compliance with national legislation following the European Union–Directive 2010/63/EU (revising Directive 86/609/EEC) for the protection of animals used for scientific purposes. The study was conducted following ARRIVE’s suggestions. The sample size required (n = 7 per group) was determined based on a power of 85%, an effect size of 0.65, and a p-value of 0.05 using the G*power program (Version 3.1.9.3).

### 2.2. Formulation preparation

Standardized turmeric extract containing 96% curcuminoid and 24% BDMC, standardized ashwagandha extract containing 20% withanolide glycoside, and standardized BSE extracts containing 85% AKBA obtained using spray drying technology were included in a polymer matrix and turned into powder. Each gram of the JHF product administered to the animals contained 45.05% total curcuminoids, 12.45% AKBA, 11.40% BDMC, and 5.31% withanolide glycosides.

### 2.3. Experimental design

After a week of acclimatization, the rats were randomly separated into four groups:

- Sedentary control group (C): Rats remained sedentary throughout the experiment without exercise and were orally administered physiological saline,
- Exercise (E) group: Rats were exercised using a rodent treadmill and orally administered physiological saline,
- E + JHF 100 group: Rats were exercised using a rodent treadmill and orally administered JHF 100 mg/kg (BDMC-enriched curcumin + Ashwagandha + BSE),
- E + JHF 200 group: Rats were exercised using a rodent treadmill and orally administered JHF 200 mg/kg (BDMC-enriched curcumin + Ashwagandha + BSE).

Before beginning the procedures, every effort was made to minimize the rats’ pain and suffering. Products dissolved in 1 mL of saline (JHF, Omniactive Health Technologies) were administered orally by gavage at 100 mg/kg and 200 mg/kg daily throughout the 42-day study period (Orhan et al., 2022).

The exercise-training program was conducted on a motorized animal treadmill (MAY-TME, Commat Limited, Ankara, Türkiye) 5 days a week, following previously established protocols. Rats began to run for 10 min/day at 10 m/min speed and a 10% grade. Over the following weeks, the duration and speed gradually increased until each rat ran continuously for 1 h/day at 27 m/in for 1 h each day for 42 days. After the training period, treadmill running until exhaustion was used to measure endurance capacity. Animals were forced to run on a motorized treadmill (5/lane) at 36 m/min speed until exhausted (Sahin et al., 2021). Exhaustion was defined as the inability of the rat to maintain an appropriate pace despite continuous hand prodding for 1 min. At this point, the rat was taken off the treadmill, and its running time was recorded. A shock grid was set up to send 0.2 mA of electricity to the animals as a warning without hurting them. After 10 s of electrical stimulation, rats were considered exhausted if unable to continue running, and their exhaustion times were noted.

The rats’ combined forefoot and hindfoot grip strengths were assessed on day 41 of the investigation. A force measurement device with a digital electronic force gauge was used to calculate the peak force. A mild grip was applied to the tail of each rat until it released the pull rod. Peak values were recorded after five consecutive tests (Sahin et al., 2021).

### 2.4. Sample preparation

Body weights (BW) were measured at the beginning and end of the study. After the exhaustion exercise, all rats were anesthetized and decapitated. Blood and gastrocnemius muscle samples were taken for analysis. The gastrocnemius muscle was put on ice and stored at −80 °C until analyzed. Tris (10 volumes of 10 mM) was used to homogenize the muscle samples for biochemical testing. Homogenates were centrifuged (10 min, 4 °C, 4000 rpm) to get the low-speed supernatant fraction.

### 2.5. Biochemical analysis

Serum triglyceride, glucose, albumin, total protein, globulin, blood urea nitrogen, total cholesterol, aspartate aminotransferase, and alanine aminotransferase values were measured using rat-specific kits with a value-determining automated biochemical apparatus (Samsung Electronics Co., Suwon, Korea). Lactate dehydrogenase (LDH, Cat No. MBS269777), serum creatine kinase (CK, Cat No. MBS267514), myoglobin (MYB, Cat No. MBS564122), and lactic acid (LA, Cat No. MBS755975) concentrations were determined by ELISA (Bio-Tek Instruments Inc., Winooski, VT, USA) following the manufacturer’s instructions. All kits were obtained from MyBioSource (San Diego, CA, USA).

Malondialdehyde (MDA) levels (muscle and serum) were measured using high-performance liquid chromatography (HPLC, Shimadzu, Tokyo, Japan) in conjunction with an ultraviolet-visible detector (UV-VIS, SPD-10 AVP, Shimadzu, Tokyo, Japan), using a C18 column (octadecyl-silica, −3.5 m, 4.6 mm 250 mm) (Orhan et al., 2022). The antioxidant enzyme activities (CAT, GSH-Px, and SOD) in muscle tissues and serum were determined using test kits (Cayman Chemical, Ann Arbor, Michigan, USA) according to the manufacturer’s instructions.

### 2.6. Protein analysis

Peroxisome proliferator-activated receptor-gamma coactivator-1 alpha (PGC-1α), interleukin-1 beta (IL-1β), IL-6, TNF-α, cyclooxygenase-2 (COX2), phosphorylated mammalian target of rapamycin (p-mTOR), P70 ribosomal protein S6 kinase 1 (p70S6K1), phosphorylated level of 4E binding protein 1 (p-4E-BP1), muscle atrophy F-box (MAFbx), and muscle RING-finger protein-1 (MuRF1) were analyzed in muscle tissue by Western blot (Sahin et al., 2021). First, the muscle tissues were pooled and homogenized with lysis buffer. The homogenate protein concentration was then measured using a Nanodrop spectrophotometer (Maestrogen Inc., Taiwan). The sample wells for sodium dodecyl sulfate-polyacrylamide gel electrophoresis (SDS-PAGE) were loaded with an identical volume of protein. The proteins were then transferred to nitrocellulose membranes (0.45 m) following electrophoresis. A 5% bovine serum albumin blocker was used to stop nonspecific protein binding. Nitrocellulose membranes were incubated overnight at 4 °C with PGC-1α, IL-1β, IL-6, TNF-α, COX2, p-mTOR, p70S6K1, p-4E-BP1, MAFbx, and MuRF-1 primary antibodies (Abcam, UK). Protein loading was controlled by the β-actin protein. A diaminobenzidine (DAB) substrate was used to demonstrate the specific binding between primary and secondary antibodies. Image J software (National Institute of Health, Bethesda, MD, USA) was used for the densitometric analysis of protein bands.

### 2.7. Statistical analysis

The data were analyzed using IBM SPSS software (IBM Corp., Version 22.0, Armonk, NY, USA). The homogeneity of variances and data normality were examined using the Levene and Shapiro–Wilk tests, respectively. To ascertain the differences between groups, one-way analysis of variance (ANOVA), and the Tukey post hoc test were used. The data are presented as the mean and standard error of the mean. Statistical significance was defined as p < 0.05.

## 3. Results

### 3.1. Exercise performance and biochemical parameters

**Figure 1:**
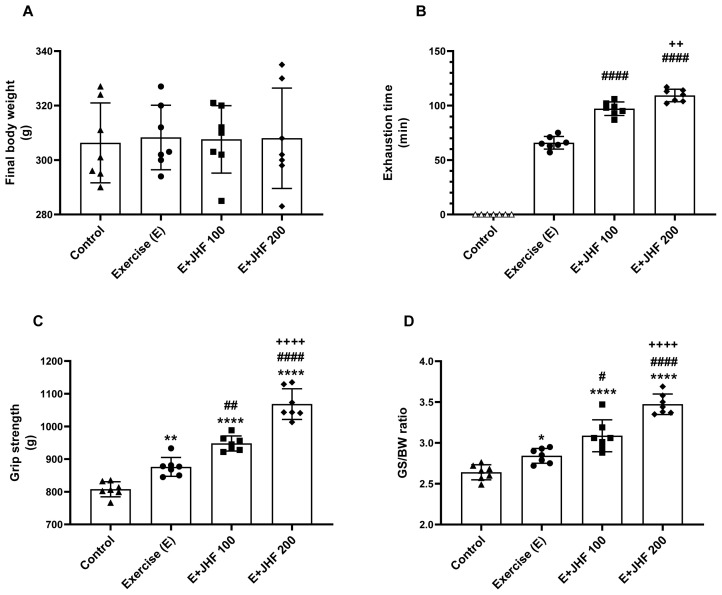
Effects of joint health formula (JHF) supplementation on body weight (A), exhaustion time (B), grip strength (C), and grip strength:body weight ratio (GS:BW; D) in exercised rats. The depicted bars represent the mean and standard deviation. Different symbols (*, ^#^, and ^+^ indicate difference compared to the Control, Exercise, and E + JHF 100 groups, respectively) above the bars indicate statistical differences among the groups (ANOVA and Tukey’s post hoc test; *p < 0.05, **p < 0.01, ****p < 0.0001; ^#^p < 0.05, ^##^p < 0.01, ^####^p < 0.0001; ^++^p < 0.01, ^++++^p < 0.0001, respectively).

Exercise and JHF supplementation did not significantly affect serum biochemical markers (p > 0.05, Table). Additionally, no difference was observed in the body weights of the rats that received JHF supplements (Figure 1A, p > 0.05).

The time to exhaustion was significantly increased by JHF treatment compared to exercise alone, particularly with the 200 mg/kg JHF dose (Figure 1B, p < 0.0001). Compared to the exercise and control groups, JHF 100 mg/kg increased grip strength (Figure 1C, p < 0.0001). Additionally, the 200 mg/kg JHF group had a greater increase in grip strength than the 100 mg/kg JHF group (p < 0.0001). The grip strength/body weight ratio was augmented in the JHF-supplemented groups compared to the control group (Figure 1D, p < 0.0001).

**Figure 2:**
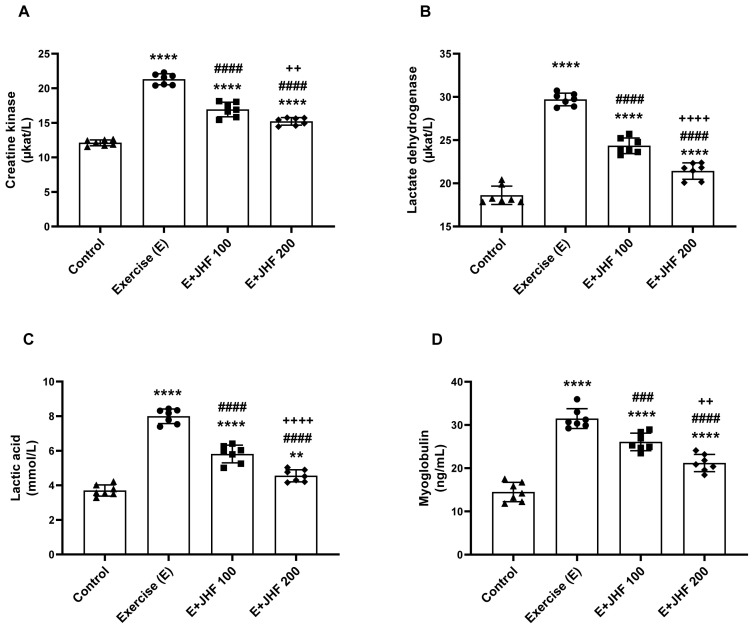
Effects of joint health formula (JHF) supplementation on serum CK (A), LDH (B), lactic acid (C), and myoglobulin (D) levels in exercised rats. The depicted bars represent the mean and standard deviation. Different symbols (*, ^#^, and ^+^ indicate difference compared to the Control, Exercise, and E + JHF 100 groups, respectively) above the bars indicate statistical differences among the groups (ANOVA and Tukey’s post hoc test; **p < 0.01, ****p < 0.0001; ^####^p < 0.0001; ^++^p < 0.01, ^++++^p < 0.0001, respectively).

Compared to the control group, exhausted rats in the JHF 200 group exhibited higher blood levels of CK, LDH, LA, and MYB (Figures 2A–2D, p < 0.0001 for all). Compared to the exercise group, the JHF 200 group blood values for CK, LDH, LA, and MYB were significantly lower (p < 0.0001 for all). However, CK (p < 0.0001), LDH (p < 0.0001), LA (p < 0.01), and MYB (p < 0.0001) values were still higher in the JHF 200 group compared to the control group. In addition, the JHF 200 group had significantly reduced blood CK (p < 0.01), LDH (p < 0.0001), LA (p < 0.0001), and MYB (p < 0.01) compared to the JHF 100 group.

### 3.2. Malondialdehyde and antioxidant enzymes

**Figure 3:**
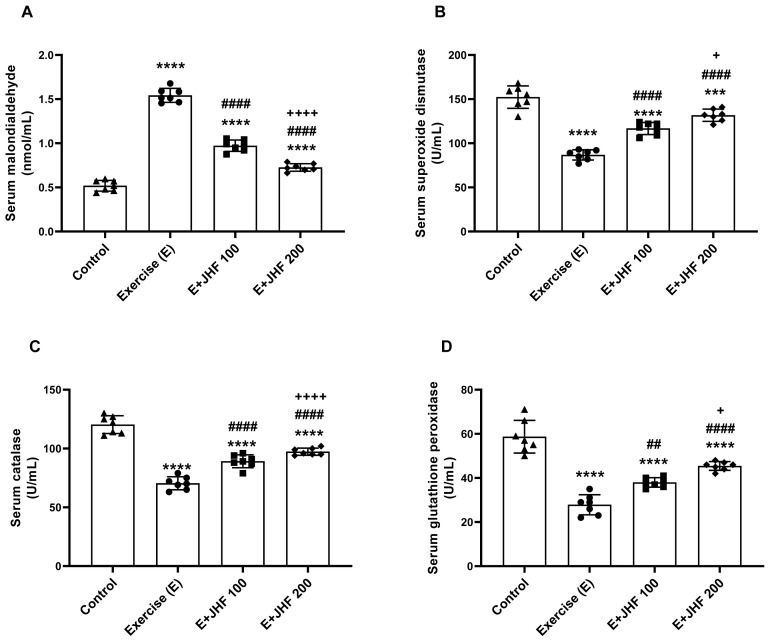
Effects of joint health formula (JHF) supplementation on serum MDA (A), SOD (B), CAT (C), and GSHPx (D) levels in exercised rats. The depicted bars represent the mean and standard deviation. Different symbols (*, ^#^, and ^+^ indicate difference compared to the Control, Exercise, and E + JHF 100 groups, respectively) above the bars indicate statistical differences among the groups (ANOVA and Tukey’s post hoc test; ***p < 0.001, ****p < 0.0001; ^##^p < 0.01, ^####^p < 0.0001; ^+^p < 0.05, ^++++^p < 0.0001, respectively).

**Figure 4:**
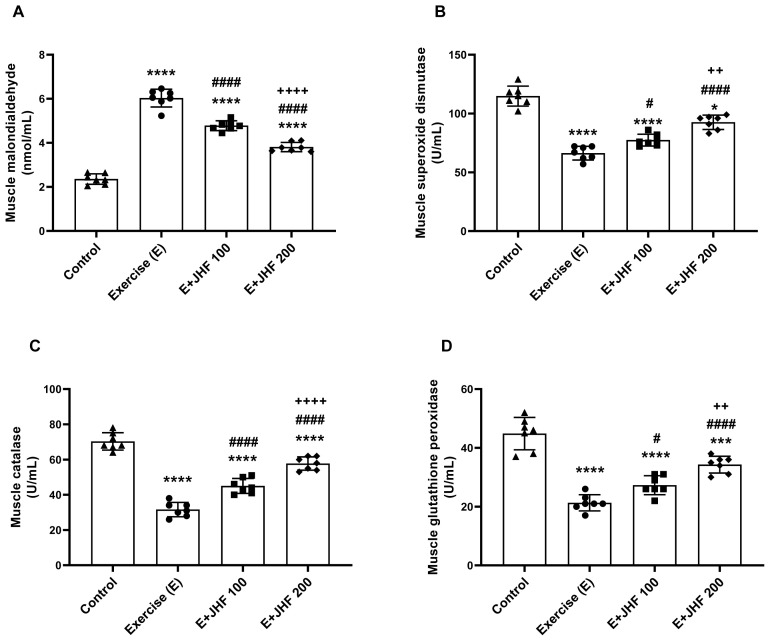
Effects of Joint Health Formula (JHF) supplementation on muscle MDA (A), SOD (B), CAT (C), and GSHPx (D) levels in exercised rats. The depicted bars represent the mean and standard deviation. Different symbols (*, ^#^, and ^+^ indicate difference compared to the Control, Exercise, and E + JHF 100 groups, respectively) above the bars indicate statistical differences among the groups (ANOVA and Tukey’s post hoc test; *p < 0.05, ***p < 0.001, ****p < 0.0001; ^#^p < 0.05, ^####^p < 0.0001; ^++^p < 0.01, ^++++^p < 0.0001, respectively).

Malondialdehyde (MDA) and antioxidant enzymes serum (Figure 3) and muscle tissue (Figure 4) results were compared with those of the other groups. Malondialdehyde **(**MDA), an important measure of oxidative stress, was significantly increased in serum (Figure 3A) and muscle (Figure 4A) in the exercise group compared to the other groups (p < 0.0001 for all). When the JHF 200 group was compared with the exercise and JHF 100 groups, the muscle and serum MDA levels were significantly reduced (p < 0.0001 for all). In contrast to MDA levels, CAT, SOD, and GSH-Px enzymes were decreased in muscle and serum after exhaustive exercise compared to the control group (p < 0.0001 for all). SOD, CAT, and GSH-Px activities were effectively increased in serum (Figures 3B–3D) and muscle samples (Figures 4B–4D) in rats supplemented with JHF (p < 0.05). Moreover, when the JHF 200 group was compared with the JHF 100 group, SOD (p < 0.05 for serum and p < 0.01 for muscle), CAT (p < 0.0001 for serum and p < 0.0001 for muscle), and GSH-Px (p < 0.05 for serum and p < 0.01 for muscle) levels were significantly increased.

### 3.3. Muscle protein levels

**Figure 5:**
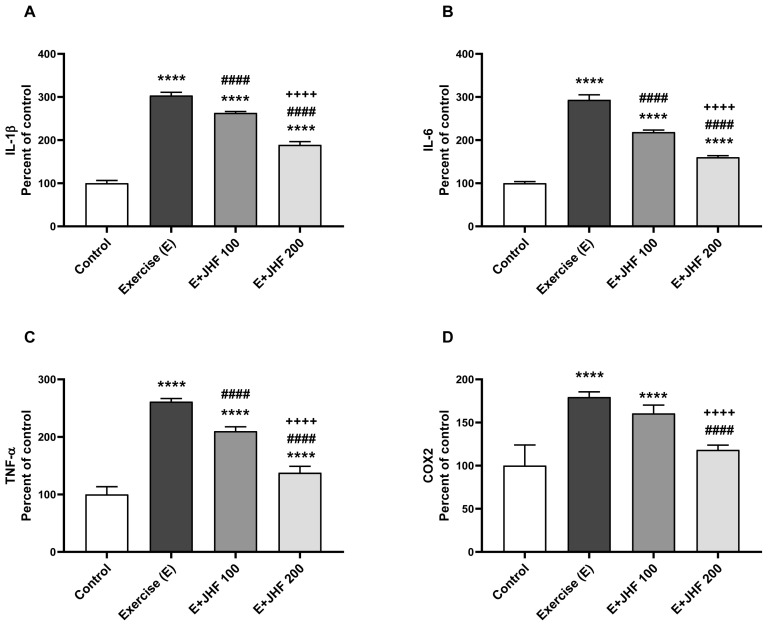
Effects of joint health formula (JHF) supplementation on muscle protein expression of IL-1β (A), IL-6 (B), TNF-α (C), COX2 (D) and PGC-1α (E) levels in exercised rats. The densitometric analysis of the relative intensity according to the control group of the Western blot bands was performed with β-actin normalization to ensure equal protein loading. Blots were repeated at least three times (n = 3) and a representative blot is shown (F). The depicted bars represent the mean and standard deviation. Different symbols (*, ^#^, and ^+^ indicate difference compared to the Control, Exercise, and E + JHF 100 groups, respectively) above the bars indicate statistical differences among the groups (ANOVA and Tukey’s post hoc test; ****p < 0.0001; ^##^p < 0.01, ^####^p < 0.0001; ^++++^p < 0.0001, respectively).

In groups that exercise exhaustion, IL-1β, IL-6, TNFα, COX2, and PGC-1α levels were significantly increased in muscle tissue, while inflammatory levels were significantly reduced in groups that received JHF supplementation (Figures 5A–5E). When the JHF 200 group was compared with the control group, the levels of muscle tissue IL-1β, IL-6, TNFα, and PGC-1α were significantly reduced (p < 0.0001), but there was no significant difference in COX2 (p > 0.05). In addition, a significant increase in the level of PGC-1α, which modulates the metabolism of muscle adaptation, was detected in exercised rats (Figure 5E; p < 0.0001). Muscle tissue IL-1β, IL-6, TNFα, COX2, and PGC-1α levels gradually decreased dose-dependently with JHF supplementation with compared exercised group (p < 0.0001).

**Figure 6:**
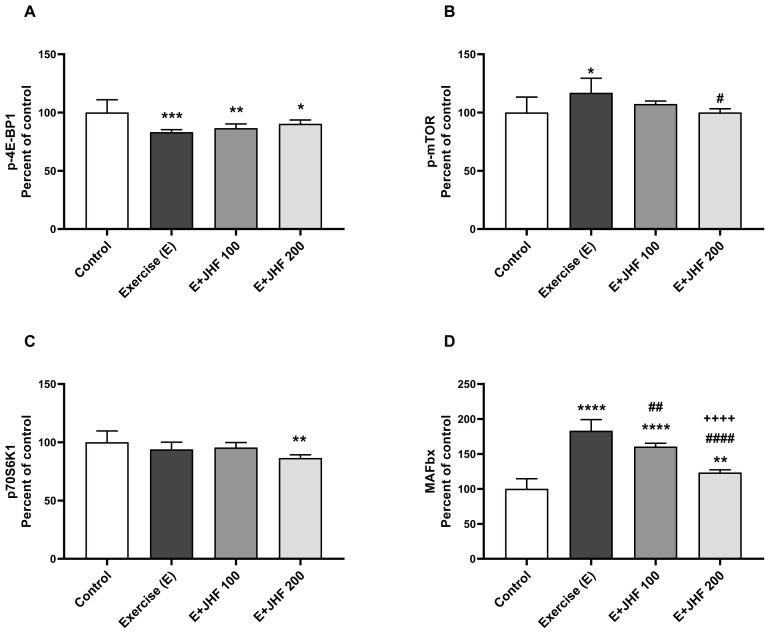
Effects of Joint Health Formula (JHF) supplementation on muscle protein expression of p-4E-BP1 (A), p-mTOR (B), p70S6K1 (C), MAFbx (D) and MuRF-1 (E) levels in exercised rats. The densitometric analysis of the relative intensity according to the control group of the Western blot bands was performed with β-actin normalization to ensure equal protein loading. Blots were repeated at least three times (n = 3) and a representative blot is shown (F). The depicted bars represent the mean and standard deviation. Different symbols (*, ^#^, and ^+^ indicate difference compared to the Control, Exercise, and E + JHF 100 groups, respectively) above the bars indicate statistical differences among the groups (ANOVA and Tukey’s post hoc test; *p < 0.05, **p < 0.01, ***p < 0.001, ****p < 0.0001; ^#^p < 0.05, ^##^p < 0.01, ^####^p < 0.0001; ^++++^p < 0.0001, respectively).

Muscle tissue levels of p-4E-BP1 decreased (Figure 6A; p < 0.001) in the untreated exercise-exhausted groups, compared with the control group (p < 0.0001). No statistical difference was detected between other groups for p-4E-BP1 (Figure 6A, p > 0.05). Moreover, p-mTOR levels increased slightly in the exercise group compared to the control group (Figure 6B; p < 0.05). However, compared with the exercise group, the level of p-mTOR protein was lower in the JHF 200 group (p < 0.05). There was a significant decrease in muscle p70S6K1 levels only in the JHF 200 group compared to the control group (p < 0.01), with no statistical difference between the other groups (Figure 6C, p > 0.05). MAFbx and MuRF-1 protein levels were significantly reduced in rats administered 200 mg/kg JHF compared to 100 mg/kg (p < 0.0001 for all). In addition, muscle MAFbx (p < 0.01) and MuRF-1 (p < 0.0001) protein levels were significantly increased in the JHF 200 group compared to the control group (Figures 6D and 6E).

## 4. Discussion

In the current study, we evaluated the therapeutic and protective effects of JHF consisting of a mixture of Ashwagandha, Boswellia (enriched with 3-O-Acetyl-11-keto-beta-boswellic acid), and curcumin (enriched with BDMC) in exercise-exhausted rats. The applied JHF doses (100 mg/kg and 200 mg/kg) were biologically safe, as they did not cause any adverse changes in biochemical parameters. In addition, supplementation with JHF reduced inflammatory and oxidative stress-induced muscle damage and fatigue while increasing grip strength and endurance capacity.

Elevated ROS levels may reduce skeletal muscle force production, possibly attributed to disrupted cytoplasmic Ca^2+^ homeostasis, muscle fiber damage, and induction of inflammation (Ma et al., 2021). A study on elderly mice determined that running endurance and grip strength increased in mice supplemented with γ-Oryzanol, which has antioxidant properties, compared to a nonsupplemented group (Ahn et al., 2021). In addition, exhaustive treadmill running has been used as an animal model of a stressful condition, as in our study, and has been shown to increase exhaustion time (Miao et al., 2021).

The production of antioxidant enzymes, such as SOD and CAT, decreases during intense exercise, but the use of antioxidant supplements can reverse this situation (Ma et al., 2021). Therefore, as in our current study, using exogenous antioxidants may help delay muscle fatigue and improve endurance exercise performance (Mason et al., 2020). In a study conducted on rats with osteoarthritis, JHF supplementation enhanced SOD, CAT, and GSH-Px production in a dose-dependent manner while significantly decreasing the amount of MDA, similar to our findings (Orhan et al., 2022).

Exercise-induced fatigue accumulates after intense exercise when LA is not eliminated promptly, leading to sports injuries. Conventional LA removal methods are limited by timeliness, metabolic load, and potential toxicity (Huang et al., 2020). In a clinical study with eccentric exercise, serum activities of CK, LDH, and aldolase were significantly suppressed in a preexercise branched-chain amino acids (BCAA) supplement group compared to a control group in the days following BCAA supplementation (Ra et al., 2018).

During the inflammatory phase following intense exercise, cytokines such as IL-6 and TNF-α are activated. TNF-α has a proinflammatory effect at the site of cellular damage and aids in muscle regeneration. It has been demonstrated that the prominent cytokine IL-6 rises during and after exercise (Markus et al., 2021). The current study prevented muscle damage in the exercise group receiving JHF, and a dose-dependent decrease in IL-1, IL-6, TNF-α, and COX-2 levels was detected.

PGC-1α is a crucial regulator of exercise-induced phenotype adaptation in muscles (Thirupathi and Souza, 2017). In addition, both protein and mRNA expression levels of PGC-1α increase following acute endurance exercise (Ray et al., 2015). Therefore, PGC-1α is predicted to be a potential regulator of metabolic adaptations after endurance exercise (Qaisar et al., 2016). In an in vivo study by Venditti et al., PGC-1α values increased with endurance exercise in rats and decreased with vitamin E supplementation (Venditti et al., 2014). These findings demonstrated the sensitivity of the PGC-1α signal to ROS activity in skeletal muscle.

A crucial nutrition sensor, mTOR is controlled by the availability of nutrients in skeletal muscle. Akt activation has been shown to trigger the mTOR/S6K1 pathway, resulting in muscle fiber hypertrophy (Liao and Xu, 2011). In recent studies, it has been reported that the phosphorylation of mTOR increases (Hayasaka et al., 2014), does not change (Majd et al., 2018), and decreases (Rivas et al., 2009) with different exercise versions. In our study, the new-generation formulation combined with exercise reduced mTOR phosphorylation. Administering JHF at 200 mg/kg further inhibited the phosphorylation rate. While p-4E-BP1 protein levels declined in the exercise group and increased following JHF supplementation, approaching the control group, p-70S6K1 protein levels decreased with 200 mg/kg of JHF compared to the control group. In a human malignant meningioma cancer cell line, treatment of cells with curcumin and tirucallic acid isolated from BSE reduced phosphorylation levels of mTOR in a concentration-dependent manner (Chilelli et al., 2016). In the current study, since JHF may have induced mTOR-dependent upregulation of PGC-1α in rats, a decreased PGC-1α level is associated with reduced mTOR phosphorylation.

In the present study, MAFbx and MuRF-1 increased in muscle tissue after endurance exercise. These results suggest that protein breakdown is triggered (Mascher et al., 2008). MAFbx and MuRF-1 knockout mice were resistant to the effects of denervation-induced muscle atrophy, with protection from muscle wasting of 56% and 36%, respectively, compared to controls (Rom and Reznick, 2016). Moreover, in an in vivo study, in line with our results, unsaturated type II collagen treatment regulated muscle metabolism and decreased MAFbx and MuRF-1 levels compared to an exercise group (Orhan et al., 2021). In the current study, a 200 mg/kg dose of JHF reduced both MuRF1 and MAFbx protein levels more effectively than 100 mg/kg. The formulation is predicted to favorably modulate levels in a dose-dependent manner in skeletal muscle.

The data showed that JHF increased antiinflammatory activity and antioxidant capacity by focusing on specific molecular pathways, thereby reducing muscle damage and increasing muscle strength and endurance in exercised rats. JHF supplementation dose-dependently modulated the mTOR phosphorylation of 4E-BP1 and PGC-1α proteins and suppressed MuRF1 and MAFbx in rats undergoing exhaustive exercise. While JHF significantly contributed to the prevention of exhaustive exercise-induced damage and improved performance in rats, human clinical research is necessary to confirm the positive impact of JHF on muscle performance and protection against muscle injury.

## Figures and Tables

**Table:** Effects of joint health formula (JHF) supplementation on serum biochemical parameters in exercised rats.

Items	Groups				p*

	Control	Exercise (E)	E + JHF100	E + JHF200	
Glucose, mg/dL	107.86 ± 3.51	110.00 ± 3.96	105.14 ± 3.47	104.71 ± 3.32	0.700
TG, mg/dL	119.29 ± 3.37	120.57 ± 2.95	118.43 ± 3.98	115.14 ± 2.52	0.681
TC, mg/dL	129.22 ± 2.82	128.49 ± 3.10	127.86 ± 2.37	125.59 ± 3.22	0.830
BUN, g/dL	19.49 ± 0.31	20.01 ± 0.25	19.25 ± 0.32	19.28 ± 0.35	0.296
TP, g/dL	7.43 ± 0.15	7.46 ± 0.14	7.47 ± 0.17	7.41 ± 0.11	0.992
ALB, g/dL	3.40 ± 0.10	3.41 ± 0.06	3.41 ± 0.06	3.43 ± 0.10	0.996
GLOB, g/dL	3.91± 0.06	3.89 ± 0.09	3.87 ± 0.08	3.86 ± 0.10	0.967
ALT, U/L	84.71 ± 2.49	85.71 ± 2.75	83.86 ± 2.94	84.00 ± 2.47	0.958
AST, U/L	109.29 ± 1.92	110.14 ± 4.16	108.43 ± 3.19	107.29 ± 2.76	0.926

TG: Triglyceride, TC: Total cholesterol; BUN: Blood urea nitrogen; TP: Total protein; ALB: Albumin; GLOB: Globulin; ALT: Alanine aminotransferase; AST: Aspartate aminotransferase. p > 0.05;

* ANOVA and Tukey’s post hoc test. Mean values of items are demonstrated with ± standard error of mean. Control (not supplement, not exercised), E: Exercise (not supplement), JHF100: Joint health formula was given at a dose of 100 mg/kg, JHF200: Joint health formula was given at a dose of 200 mg/kg.
